# The Current Research Landscape on the Machine Learning Application in Autism Spectrum Disorder: A Bibliometric Analysis From 1999 to 2023

**DOI:** 10.2174/011570159X332833241222191422

**Published:** 2025-03-25

**Authors:** Xinyu Li, Wei Huang, Rongrong Tan, Caijuan Xu, Xi Chen, Qian Zhang, Sixin Li, Ying Liu, Huiwen Qiu, Changlong Bi, Hui Cao

**Affiliations:** 1 Department of Psychiatry, The School of Clinical Medicine, Hunan University of Chinese Medicine, Changsha, Hunan, China;; 2 Department of Psychiatry, Brain Hospital of Hunan Province (The Second People’s Hospital of Hunan Province), Changsha, Hunan, China;; 3 Department of Integrated Traditional Chinese and Western Medicine, Xiangya Hospital, Central South University, Changsha, Hunan, China;; 4 Department of Neurosurgery, Xiangya Hospital, Central South University, Changsha, Hunan, China;; 5 National Clinical Research Center for Geriatric Disorders, Xiangya Hospital, Central South University, Hunan, China

**Keywords:** Autism spectrum disorder, machine learning, bibliometric analysis, Hot-spots, Citespace, VOSviewer

## Abstract

**Background:**

Language deficits, restricted and repetitive interests, and social difficulties are among the characteristics of autism spectrum disorder (ASD). Machine learning and neuroimaging have also been combined to examine ASD. Utilizing bibliometric analysis, this study examines the current state and hot topics in machine learning for ASD.

**Objective:**

A research bibliometric analysis of the machine learning application in ASD trends, including research trends and the most popular topics, as well as proposed future directions for research.

**Methods:**

From 1999 to 2023, the Web of Science Core Collection (WoSCC) was searched for publications relating to machine learning and ASD. Authors, articles, journals, institutions, and countries were characterized using Microsoft Excel 2021 and VOSviewer. Analysis of knowledge networks, collaborative maps, hotspots, and trends was conducted using VOSviewer and CiteSpace.

**Results:**

A total of 1357 papers were identified between 1999 and 2023. There was a slow growth in publications until 2016; then, between 2017 and 2023, a sharp increase was recorded. Among the most important contributors to this field were the United States, China, India, and England. Among the top major research institutions with numerous publications were Stanford University, Harvard Medical School, the University of California, the University of Pennsylvania, and the Chinese Academy of Sciences. Wall, Dennis P. was the most productive and highest-cited author. Scientific Reports, Frontiers In Neuroscience Autism Research, and Frontiers In Psychiatry were the three productive journals. “autism spectrum disorder”, “machine learning”, “children”, “classification” and “deep learning” are the central topics in this period.

**Conclusion:**

Cooperation and communication between countries/regions need to be enhanced in future research. A shift is taking place in the research hotspot from “Alzheimer's Disease”, “Mild Cognitive Impairment” and “cortex” to “artificial intelligence”, “deep learning”, “electroencephalography” and “pediatrics”. Crowdsourcing machine learning applications and electroencephalography for ASD diagnosis should be the future development direction. Future research about these hot topics would promote understanding in this field.

## 1 INTRODUCTION

Typically, autism is characterized by a lack of language proficiency, restricted and repetitive interests, and social difficulties [1]. In 1943, child psychiatrist Kanner first described ASD based on 11 children's characteristics [2]. It is estimated that more than 3/4 of ASD patients have many mental diseases, such as deficiency of intellect, attention deficit hyperactivity disorder (ADHD), and sleep disorder, it is the leading cause of mental disability among children under 5 years old [3]. There has been an increase in the prevalence of autism spectrum disorder among children aged 8 years in the United States from approximately 1.1% in 2008 to 2.3% in 2018 [4]. Rather than a condition caused by the impairment of a focal impairment in a specific brain region or system, ASD is now understood to be the result of an overall reorganization of the brain that occurs early in life [5]. Individuals, their families, and society suffer a greater economic burden due to the lack of specific diagnostic markers and targeted treatment options for ASD [6]. Detecting ASD early is critical to developing effective early interventions that can improve long-term outcomes and ameliorate defining deficits.

In recent years, treatments for autism spectrum disorder have seen significant advancements in artificial intelligence (AI). An important part of AI is machine learning, which enables computers to learn from data. AI commercial applications are driven by machine learning, which is an overarching term for several different methods of achieving AI. These applications are all a result of a variety of machine-learning methods that enable machines to learn [7]. In the field of medicine, machine learning has proven to be a valuable tool. In recent years, these methods have been shown to be effective diagnostic and predictive tools for identifying a variety of diseases [8]. The application of machine learning to structural and functional neuroimaging data has been successful in treatment prognosis, transition prediction and disease diagnosis [9]. It was also utilized in combination with neuroimaging techniques to study the ASD in order to find out its neurophysiological basis [9, 10]. Machine learning has a promising future in medicine [11].

The two principal advantages of bibliometrics are its domain independence and its capacity to analyze large quantities of publications [12]. In medicine, bibliometrics is used to analyze large amounts of publications on a macroscopic and microscopic level [13]. Bibliometric analysis was a statistical examination that analyzed scientific publications *via* bibliometric tools, such as CiteSpace and VOSviewer [14], to define the literature landscape and future trends [15]. A number of disciplines have applied CiteSpace to related research because it is capable of supporting multiple data formats, offers comprehensive functions, and provides good visualization effects. An analysis of the overall application status and research trend of AI in ASD has been conducted in recent years based on bibliometric data. A bibliometric analysis article discovered wearable devices and brain-computer interfaces are the direction of further exploration of AI in ASD [16]. However, these articles are not centered on machine learning. Although a significant number of bibliometric analyses dealt with ASD and machine learning respectively, the application of machine learning in ASD has yet to be systematically reviewed and bibliometrically analyzed in the medical literature, as far as we know. Therefore, this study aims to examine the use of machine learning in ASD, including research trends and the most popular topics, as well as proposed future directions for research.

## 2 METHODS AND MATERIALS

### 2.1 Search Strategy

On January 7, 2024, the search query string was used to conduct an advanced search on WoSCC, TS = (“Machine learn*” or “Robotic learn*” or “Transfer learn*” or “Deep learn*” or “machine AND learning”) and TS = (“Autism disorder” or “Autistic disorder” or “Autistic spectrum disorder” or “Autism spectrum disorder” or “Autism” or “ASD”), to identify publications related to machine learning in autistic disorder. We only accepted articles and reviews, and only allowed English-language documents. For this review, the database was independently searched by Xinyu Li, who screened titles and abstracts and eliminated articles. A consensus was reached by discussing discrepancies with Changlong Bi and Hui Cao.

### 2.2 Data Extraction and Analytical Methods

WoS stores high-quality publications based on three main indices: Science Citation Index Expanded, Social Sciences Citation Index, and Arts & Humanities Citation Index. Using the WoSCC database, we exported all publications and their citations. Citation counts, citations per paper, H-index, and references, along with title, country, keywords, journal, institution, publication year, and author, were extracted as bibliometric parameters. We imported these data into Microsoft Excel 2021 (Redmond, Washington, USA), VOSviewer (Leiden University, Leiden, Netherlands), and two online platforms (https://flourish.studio/examples/ and https://bibliometric.com/) to identify the most prolific contributors (authors, institutions, and countries). Scimago Graphics is a software program designed to facilitate the creation of charts illustrating the geographic distribution of literature and the publication trends associated with it.

There is a positive correlation between the size of the nodes and the number of articles in VOSviewer. The co-authorship analysis assessed collaboration among authors, countries, and institutions [17]. The total link strength (TLS; the sum of all links connected to a node) represents the extent to which it cooperates with other nodes. A positive correlation exists between link width and cooperation strength between two nodes. VOSviewer (Version 1.6.11) and CiteSpace (Version 6.2.R6) were used to visualize keyword co-occurrence analysis and reference analysis. VOSviewer and CiteSpace's maps show nodes representing publishers and lines representing links between them. Larger nodes represent more publications, while thicker lines indicate stronger cooperation between nodes [18]. Using CiteSpace can visualize citations in the scientific literature in order to understand and track knowledge generation. In CiteSpace, keyword bursts and reference bursts could be displayed [19].

## 3 RESULTS

### 3.1 General Data

**Fig. (2):**
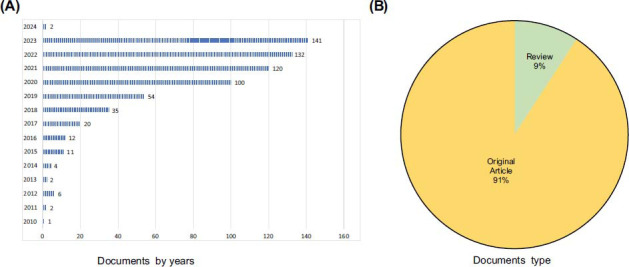
Years (**A**) and types (**B**) of publications.

Fig. (**1**) shows the design and analytical approach of the study. The flow diagram indicates every step of the search strategy and the amount of different publications. From 1999 to 2023, we retrieved 1357 publications/papers. After limiting the type of literature to original research and reviews written in English, 642 articles were retrieved. As of the search date, total citations (TC), citations per publication (CPP), and H-index reached 14358, 22.36, and 58 respectively. In total, 3064 authors, 1276 institutions, 64 countries/territories, and 46 journals contributed to this publication. In general, there were three phases of publication variation from 1999 to 2023, including the first phase (2010-2012), and the second phase 2 (2013-2023). The publication grew slowly from 1 to 6 in the first phase, and the number in the second phase remained steady from 2 to 141. The publication reached a peak of 141 in 2023 (Fig. **2A**). Review articles make up 9% of the retrieved publications, while original articles account for 91% (Fig. **2B**).

### 3.2 Top Contributing Authors

Table **1** presents the top contributing authors in machine learning in autistic disorders. The most prolific author was Wall, Dennis Paul from Stanford University (USA) with 22 publications. He was followed by Thabtah, Fadi from the University of Huddersfield (New Zealand) with 12 publications, and Elbaz, Ayman (University of Louisville, USA) and Washington, Peter (Stanford University, USA) with 9 publications. VOSviewer was used to analyze the author's cooperation network with a minimum of 20 publications. Ultimately, 20 authors with more than three articles are visualized in Fig. (**3A**), showing that Wall, Dennis P (USA) was the first-tier author with 84 TLS, followed by Washington, Peter (USA) with 69 TLS. In Fig. (**3B**), it was found that Wall, Dennis Paul from Stanford University, USA was the author with the most citations (1066 TC and 48.45CPP), followed by Mechelli, Andrea(757 TC and 378.5CPP). Wall, Dennis Paul was the center in collaboration with Washington, Peter, indicating the reason for his high citations. They both work for Stanford University.

### 3.3 Top Contributing Institutions

Based on the findings of this study, Table **2** lists the most prolific institutions. With 26 publications, Stanford University (USA) ranks first. Harvard Medical School (USA) has 21 publications, the University of California (USA) has 21 publications, and the University of Pennsylvania (USA) has 16 publications. According to citations, there are 1407 citations at the University of Pennsylvania (USA) ranked first, 1018 citations at the University of London (UK), and 992 citations at the Children’s Hospital Philadelphia (USA). With VOSviewer and CiteSpace, the cooperative network of the institution was visualized. Fig. (**4A**) illustrates 28 institutions with at least 7 documents. Stanford University (USA) and Harvard University (USA) were the central nodes in Northern America. University College London (England) and the University of Cambridge made outstanding contributions to Europe. In addition, CHINESE ACAD SCI and Beijing Normal University were the centers in China. The results suggested that a typical region feature had characterized inter-institutional cooperation. Fig. (**4B**) displays the top 15 most active funding agencies in machine learning in ASD. The United States Department Of Health and Human Services is the most funded organization, followed closely by the National Institutes of Health (NIH), USA.

### 3.4 Top Contributing Countries/Regions

**Fig. (5):**
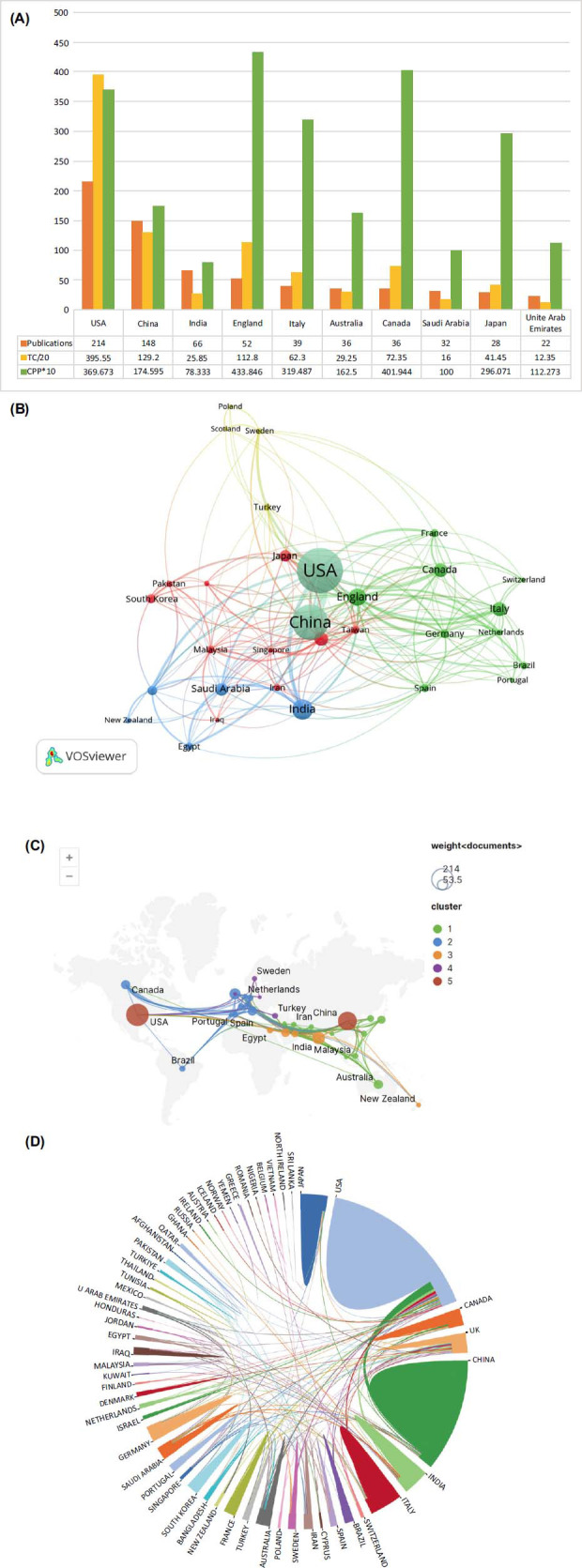
(**A**) This data includes the number of publications, total citations (×0.05), and citations per publication (×10) for each of the top ten prolific countries. (**B**) Cooperation between countries. Documents are represented by the size of nodes. A link's width indicates how strong the cooperation is. (**C**) The collaboration of countries. The size of the node indicates the number of articles produced. Links with a wide width indicate a strong cooperation. (**D**) Collaborations across countries and regions visualized. A nation's boundary line indicates the extent of collaborative interactions between nations.

In Fig. (**5**), you can see which countries are most productive and how they collaborate. It appears that 64 countries/regions actively contributed to the study of machine learning in ASD, based on a comprehensive analysis as depicted in Fig. (**5A**). The United States dominates the field, publishing 214 papers (33.3% of the total) and citing 7,911 papers (59.4%). Second place went to China with 148 publications and 2584 total citations, while third place went to India with 66 publications and 517 total citations (Fig. **5A**). The VOSviewer's co-authorship-country analysis indicates a degree of collaboration. 31 countries were selected for the visualization with at least five publications. In the visualization, the USA, China, England, India, and Canada are the most prominent nodes with relatively thicker connections, indicating that they collaborate more closely in this area and have a greater impact on academic research (Fig. **5B**). It is clear from Figs. (**5C** and **5D**) that the United States, the United Kingdom, Canada, India, and China are the most interconnected and closely collaborated countries in the field.

### 3.5 Top Contributing and Co-cited Journals

Table **3** lists the top 10 active journals publishing articles related to ASD in machine learning, which are ranked Q1, Q2 or Q3 by journal citation report (JCR). At the top is the Scientific Reports (n = 24), followed by Frontiers In Neuroscience (n = 22) and the Autism Research (n = 19) and Frontiers In Psychiatry (n = 19). Table **4** lists the top 10 co-cited journals. In terms of co-citation, the Neuroimage ranks first (n = 986), followed by the Neuroimage-Clinical (n = 885) and Translational Psychiatry (n = 777). However, Fig. (**6**) lists the top 10 most cited journals. The highest citation journal is the Science (TC = 706) and has recorded the highest CPP (n =353), followed by Neuroscience And Biobehavioral Reviews (TC = 691, CPP = 345.5). However, Fig. (**6**) lists the top 10 most cited journals. The highest citation journal is the Neuroimage (TC = 986) and Nature has recorded the highest CPP (n = 601), followed by Neuroimage-Clinical (TC = 885, CPP = 98.3333)and Translational Psychiatry (TC = 777, CPP = 59.7692).

### 3.6 Top Cited Articles

A list of the 10 most cited publications is presented in Table **4**. In Neuroscience and Biobehavioral Reviews by 2012, Orrù Graziella *et al* had 701 citations, which was the article with the most citations. The article entitled “Using Support Vector Machine to identify imaging biomarkers of neurological and psychiatric disease: A critical review”. The authors combined Neuroimaging and Support-Vector-Machine (SVM) to examine autistic spectrum disorders. Additionally, Neuroimaging data can be used as input to SVM to help diagnose neurological and psychiatric disorders [9]. The second most cited article (601 TC) was produced by Hazlett *et al.* in Nature. In this study, MRI images of infants' brains were used to feed a deep-learning algorithm. Autism diagnosis could be predicted in individual high-risk children [20]. Arbabshirani Mohammad R. and colleagues wrote a review article that received the third-highest number of citations (522TC) and was accepted by Neuroimage. This is a review of neuroimaging-based subject prediction of brain diseases, including ASD [21].

### 3.7 Analysis of Co-citation References

**Fig. (7):**
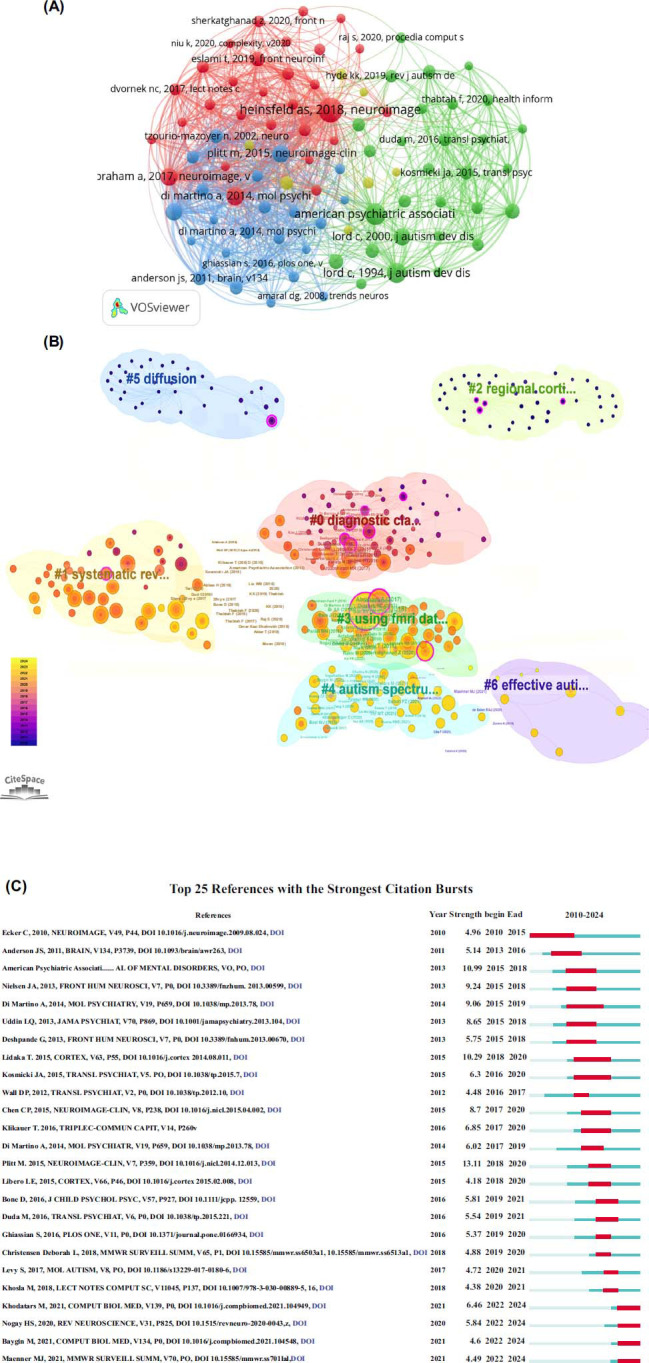
(**A**) Machine learning and autism co-citation network visualization. (**B**) CiteSpace clustered co-citation network of references. There are different colors for the nodes and links. An earlier cocitation relationship is represented by dark color. Network nodes named by the first author (publication year) display the top 10% of co-cited references. There is a positive correlation between node size and citation number. Based on the Citespace LLR algorithm, the cluster name was automatically identified. (**C**) A list of the 25 strongest burst references. The Burst duration is represented by the red bar. It is important to the research field because of its burst.

The 642 retrieved publications cited 8,255 references. In Fig. (**7A**), a visualization map of co-citations is shown. The paper titled “Identification of autism spectrum disorder using deep learning and the ABIDE Dataset” published in Neuroimage-Clinical in 2018 was the most cited reference [22]. To discover the evolution of scientific paradigms in machine learning in ASD, CiteSpace was used to analyze co-citations. The network displays articles with at least 8 citations, as shown in Fig. (**7B**). Popular themes in most co-cited references from 2018 to 2022 included “deep learning” “automatic detection” “artificial intelligence” and “automatic autism spectrum disorder detection”. “Automatic autism spectrum disorder detection” was the main topic in the next decade. In addition, we identified significant references to this field's knowledge by using a citation burst. By using CiteSpace, CiteSpace identified the top 25 publications [1, 4, 23-49] with the greatest burst of citations (Fig. **7C**). The article written by Plitt *et al*. with the highest citation bursts (n = 13.11), as a diagnostic biomarker for autism, resting-state functional MRI connectivity (rs-fMRI) was evaluated. Statistics indicate that RS-fMRI scans alone can accurately classify individuals as having ASD, but does not meet biomarker standards [25]. Diagnostic of statistic manual of mental disorders: DSM-5 (5th edition) with the second-highest citation bursts (n = 10.99). In its original form, DSM-5 was intended to be used as a guide for clinical practice. Using the ICD-10 model, it dispenses with clinician-optimized and researcher-optimized versions of the classification, making these dimensional assessments available to researchers as well as quantitatively minded clinicians [1]. The article, written by Tetsuya Iidaka with the third-highest citation bursts (n = 10.29) was published in Cortex on februay 2015. Using rs-fMRI data, the researchers developed a matrix to measure intrinsic connectivity, which may serve as a biomarker for ASD and contribute to its neurobiology [35].

### 3.8 Analysis of Keywords

**Fig. (8):**
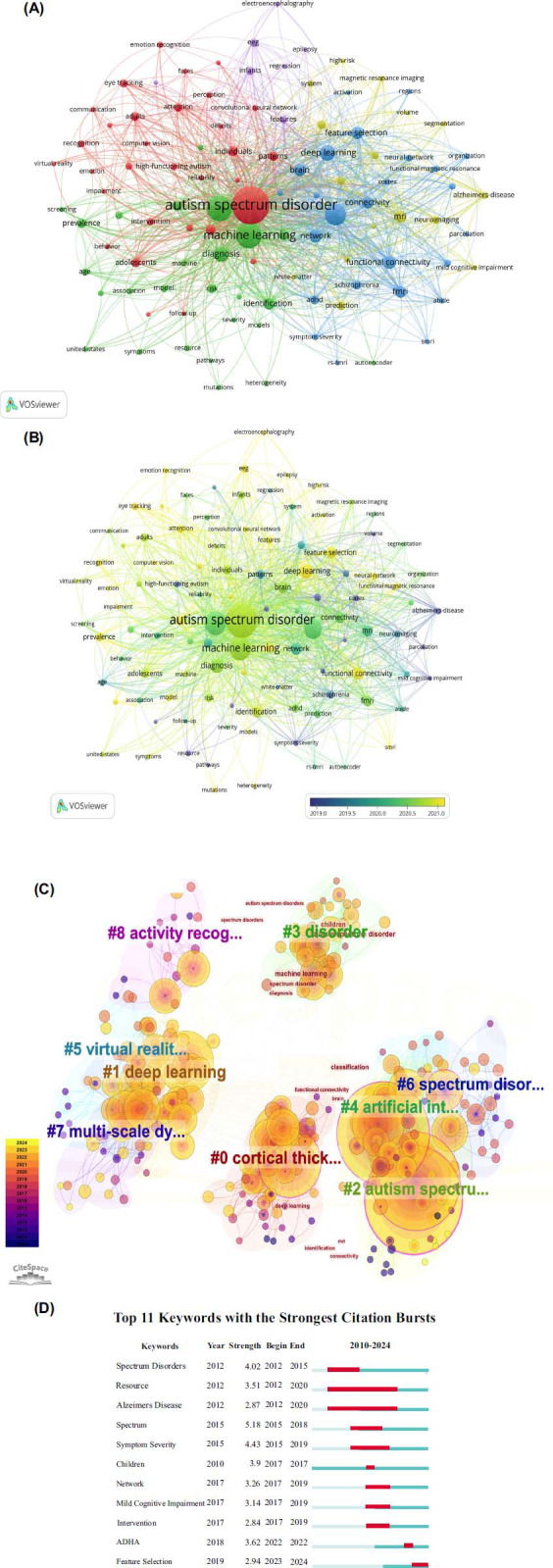
The keyword analysis. (**A**) VOSviewer visualizes keyword co-occurrence networks. The larger the node, the more frequent the keyword; a closer relationship is indicated by the same color. (**B**) A time-series analysis of keyword co-occurrences. Yellow indicates the latest appearance, and dark blue indicates earlier appearances. (**C**) Between 1999 and 2023, CiteSpace LLR named keyword clusters. (**D**) Citation bursts for the top 11 keywords. Burst duration is indicated by the red bar. This article's importance to the research field can be seen in the strength of the burst.

With the help of VOSviewer and CiteSpace, a keyword co-occurrence analysis was performed to identify major themes and potential research trends within this field. Furthermore, we merged these keywords with similar meanings using a thesaurus . For example, “autism” and “autism spectrum disorders” and “spectrum disorders” were replaced by “Autism Spectrum Disorder”. A total of 2,496 keywords were identified by VOSviewer. Only keywords that occurred more than 8 times were visualized in the co-occurrence network. As a final step, 105 keywords were clustered into five groups (Fig. **8A**). The top 10 most frequently occurring keywords are as follows: “autism spectrum disorder” (n = 494), “machine learning” (n = 235), “children” (n = 226), “classification” (n = 161), “deep learning” (n = 88),”diagnosis” (n = 79), “functional connectivity” (n = 59), “network” (n=56), “feature selection” (n = 55) and”brain” (n = 53)”. We also used VOSviewer and Citespace timeline views of keyword co-occurrence analysis to identify potential future research directions in this field as shown in Figs. (**8B** and **8C**). A color scheme was used to indicate the average year of publication for keywords. Dark colors showed seminal keywords in the early stages, including “Alzheimer's Disease”, “schizophrenia”, “Mild Cognitive Impairment” and “cortex”. The recent popular keywords were represented with light colors, namely “artificial intelligence”, “deep learning”, “eye tracking”, “electroencephalography”, “functional magnetic resonance imaging”, “pediatrics”. CiteSpace's burst module can detect keywords frequently mentioned during a certain time period [50]. As displayed in the top 11 keywords (Fig. **8D**), “spectrum” has the highest burst strength (n = 5.18), followed by “spectrum disorders” (n = 4.02) and “children” (n = 3.9).

## 4 DISCUSSION

### 4.1 Author Analysis

Wall, Dennis P. from Stanford University (USA) is the top 1 authors with high publications. Wall, Dennis P. and his team used machine learning techniques to enhance the effectiveness of clinical diagnostics, leading to reduce the time it takes to screen and intervene with autistic people [50, 51]. In their study, they concluded that machine learning analysis enables faster diagnosis without sacrificing accuracy [52, 53]. The emerging field of human-in-the-loop and crowdsourcing for autism diagnostics is being championed by Wall, Dennis P. Machine-learning algorithms can detect developmental delays in children using crowdsourced, privacy-protected analysis of short home videos [54]. It is possible that crowdsourcing machine learning may improve raters' ability to recognize and measure phenotypic manifestations of autism [55-57]. Assessment of motor imitation in children with autism using a single camera significantly associated with the severity of ASD symptoms [58]. It may providing translational solutions that might assist clinical practice [59]. Autism behavior can be detected automatically using computer vision to assist clinicians and parents [60, 61]. Wall, Dennis P. was the center in collaboration with Washington, Peter, indicating the reason for his high citations. Apart from the authors mentioned in Table **1**, Guillermo Sapiro, James Rehg, Geraldine Dawson, Rosalind Picard and Brian Scassellati *et al*. also are prominent in the field of autism and machine learning.

### 4.2 Country and Institution Analysis

This study was based on documents about ASD with machine learning in 1191 institutions from 64 countries/regions, indicating that the interest in this area was worldwide. There are eight developed countries on this list and two developing countries (China and India) listed. In developed countries, people put more emphasis on their health and wellness than in developing countries. Additionally, developed countries may devote more resources to the research the application of machine learning in ASD. In Asia, China and India are the two most populous developing countries. As a result, the study argues that ASD should be more extensively studied by machine learning in these countries. The USA, as the most prolific country, was predominant in the field, 214 publications (33.43% of the total) and 7,911 total citations. The social burden of ASD resulted in a significant number of publications in the USA. The USA has devoted a significant amount of money, manpower, and materials to early diagnosis of ASD. A leading source of studies shows that autism patients in the United States are better served by policies ranked second with 148 publications and 2,584 total citations. In 2014, ASD patients in China's cities were asked to receive clinical diagnosis as well as treatment. Approximately 1.6 billion Chinese yuan have been invested in ASD research by the National Natural Science Foundation of China since 2010 [63]. In addition, the majority of the most prolific institutions came from the United States (7/11 institutions). Stanford University, Harvard medical school, University of California, and University of Pennsylvania collaborated, explaining why the publications of USA led the head. International collaboration centers have historically been centered in the USA, which is known for its high productivity and central location. In both scientific and academic research, the United States emerged as a dominant force. The active funding agencies and the collaboration between talents in the famous university, explaining why the publications of USA led the head.

### 4.3 Journal Analysis

In this study, all 10 of the most prolific journals ranked in the highest qualities in the 2022 JCR. A journal's quality was also assessed using CPP in addition to its number of publications. Only 5 journals in the top 10 productive journals were the journals with high citations, suggesting that the other five journals with high publications should improve the standard for publication. Consequently, Table **3** are the 10 most classic and influential journals in this field. such as the Scientific Reports and Frontiers In Neuroscience. Researchers could benefit from knowing which prolific journals to submit articles to and how to gain a deeper understanding of new topics if they are familiar with prolific journals. The publication of articles in co-citation journals could also contribute to the development of future literature.

### 4.4 Hotspots and Research Trends

In order to conduct a high-quality scientific investigation, it is imperative that a scientific network is extensively evaluated. By analyzing keywords and references, a comprehensive scientific network could be visualized and the interaction between topics could be visualized [64, 65]. Through the use of VOSviewer and CiteSpace in this study, the scientific network of machine learning application in the management of ASD was visualized in terms of notable keywords and seminal references. In this particular field, the topics investigated have gradually changed. For example, in 2018, documents were centered on “Cognitive Impairment”, “Alzheimer's Disease”. In 2020, manuscripts focused on “Autism Spectrum Disorder”, “Machine Learning”, “brain connectivity”, “MRI”, and “Diagnosis”. Machine learning in medical fields has been extensively studied [66], the medical imaging developed of a novel machine learning algorithms the same time [67]. While in 2021, the topics changed to “artificial intelligence”, “deep learning”, “machine learning”, “electroencephalography” and “pediatrics”. “Artificial intelligence” is the hotspot keyword with the high burst intensity, and more and more studies have shown that artificial intelligence can help improve the emotional cognition ability of ASD patients [68]. And a growing number of studies analyze electroencephalography signals to diagnose autism [69]. Machine learning treatment of ASD is projected to continue to show an overall increase in the coming years, according to current trend analysis. The following section extensively evaluated the keywords and citations with cluster creation.

### 4.5 Cluster 1 (Red in Fig. 8A): The use of Machine Learning in Different ASD Individuals

The primary keywords were “autism spectrum disorder”, “individuals”, “adolescents”, “patterns”, “adults”, “eye tracking” and “high-functioning autism”. The prevalence of ASD around the world is approximately 1/100 children [70]. Socialization, communication, and repetitive or unusual behaviors are common characteristics of ASD [71]. It would be beneficial to use predictive tools in order to facilitate personalized diagnosis, prognosis, and treatment selection for children and adolescents [72]. In addition, uncovering brain differences in children and young adolescents with ASD by using deep learning [73]. A research examined gaze-contingent eye tracking's feasibility, acceptability, and effectiveness in targeting emotion recognition in youth with autism [74].There are many manifestations of autism spectrum disorder, making its diagnosis challenging, especially for highly independent adults. Researchers used gaze data from web-related tasks to develop a machine-learning classifier that can detect autism in adults [75]. Adults with high-functioning autism can be detected automatically with eye-tracking data and machine learning [76].

### 4.6 Cluster 2 (Green in Fig. 8A): Machine Learning in the Diagnosis of Children with ASD

The primary keywords were “machine learning”, “children”, “diagnosis”, “identification”,“expressions” and “artificial intelligence”. Autism is a complex neurodevelopmental condition that can be challenging to diagnose accurately due to the variability in associated symptoms and severity. Machine learning is widely applied to the diagnosis of ASD in children. A framework integrates advanced computer vision and machine learning strategies to computationally analyze how children with autism and typically developing children express themselves. The method could be utilized to analyze the emotional competence of young children with autism disorders [77]. Researchers in the fields of computer vision and machine learning can use machine learning to build algorithms that can recognize facial expressions of emotion [78]. ASD diagnosis and treatment can be made easier and faster through AI and machine learning, with in-home therapeutic approaches designed for all ages [79]. It has been shown that touch screen mobile computers are effective in facilitating autistic children's learning [80]. At-home therapy children with ASD use a system for automatic facial expression recognition *via* superpower glass [81, 82]. Wearable digital intervention for ASD children demonstrated efficacy in improving their social behavior [83-85]. Medical clinics and diagnosticians will benefit greatly from a new artificial intelligence-based autism screening system, and it may revolutionize the way ASD is diagnosed in the future [86, 87]. Research on human behavior and clinical translation are made possible by machine learning [41].

### 4.7 Cluster 3 (Blue in Fig. 8A): The Application of Machine Learning for Distinction of Autism and ADHD

The primary keywords were “classification”, “deep learning”, “functional connectivity”, “fmri”, “feature selection” and “adhd”. Many studies developed the novel machine learning algorithm by neuroimaging [68] and magnetic resonance imaging (MRI) [88]. MRI provides a non-invasive way to measure macroscopic functional connections Physicians place a great deal of importance on MRI imaging modalities. For accurate diagnosis of ASD, clinicians rely on MRI modalities. In addition, ASD is typically diagnosed using deep learning schemes. Machine learning technologies and MRI neuroimaging are expected to contribute to the diagnosis of ASD and helping clinicians soon [88, 89]. Even though resting-state functional MRI (rs-fMRI) scans can be used to diagnose ASD with statistically significant accuracy, this method falls short of biomarker standards [25]. The use of classification methods provided additional evidence that large-scale networks are dysfunctional in ASD functional connectivity [25]. A structural and functional MRI prediction model for ADHD and autism has been developed [90] and the use of machine learning can applied to for behavioral distinction of autism and ADHD [42]. There is a dire need to explore deep learning in the diagnosis of ASD. Deep neural network seems to be more effective in the diagnosis of autism disorder [91]. fMRI provides a promising way to facilitate diagnosis.

### 4.8 Cluster 4 (Yellow in Fig. 8A): Brain Abnormalities of ASD Patients

The primary keywords were “abnormalities”, “prediction”, “support vector machine” “cortical thickness”, “neuroimaging” and “Alzheimer’s-disease”. With the use of structural and functional neuroimaging data, Support Vector Machines (SVM) have been successfully used to diagnose disease, predict transitions and predict treatment outcomes [9]. What’s more, SVM has a better ability to distinguish whether autistic children are combined with intellectual disability [92]. In ASD, the brain's neural activity can be affected. Currently, computer-aided brain disease diagnosis is utilizing machine learning approaches [93]. In ASD patients, morphological anomalies are identified by computer-aided diagnostics (CAD). It could identify ASD associated cortical markers and the extraction of cerebral cortex from structural MRI [94]. Researchers have found that cortical thickness plays a role in the etiopathogenesis of ASD core symptoms. Biomarkers play an important role in diagnosing ASD. Squarcina *et al*. apply SVM to identify specific cortical thickness alterations in ASD subjects [95]. Moradi *et al*. predict symptom severity based on cortical thickness measurements with ASD from four different sites [96]. Corpus callosum abnormalities were mostly limited to diffusion tensor imaging (DTI). In the corpus callosum, the most striking differences in edge density appear to be related to ASD [97]. In order to detect autism spectrum disorders, the corpus callosum and the volume of the intracranial brain are critical indicators. Machine learning was used to detect ASD based on corpus callosum and intracranial brain volume features proposed by Sharif [98].

### 4.9 Cluster 5 (Purple in Fig. 8A): EEG for Autism Diagnosis

The primary keywords were “eeg”, “infants”, “pediatrics”, “epilepsy”, “electroencephalography”. Electroencephalography (EEG) signals are widely used to record brain activity. Autism diagnosis relies primarily on behavioral tests and interviews. However, in recent years, there has been an increase in studies analyzing EEG signals for autism diagnosis. According to a study, the resting-state EEG microstate analysis technique can predict autistic traits to a certain extent [69]. In different discriminative powers, EEG may be the most effective means of discrimination for the detection of ASD and typically developing children [99]. According to long EEG signals, Ardakani *et al*. could detect ASD subjects with 100% accuracy [100]. Based on a systematic review, EEG and other machine learning methods are useful for diagnosing high-risk infants as early as 3 months of age with ASD, classifying the disorder, and predicting its diagnosis [101]. ASD diagnosis can be predicted through EEG in infancy.

## 5 LIMITATIONS

In this study, several limitations were identified. At first, because of the data format required for VOSviewer and CiteSpace, the manuscripts about machine learning application in ASD were searched from a single database (WOSCC), leading to selection bias. Data from other sources, such as PubMed, could retrieve the comprehensive literature with high quality in this field but should be merged to become compatible with VOSviewer and CiteSpace. As a result, we employed two bibliometric tools (CiteSpace and VOSviewer) to reduce selection bias and eliminate duplicate literature from multiple sources. In addition, this study only included articles in English, so there may be language bias. It would be beneficial to incorporate publications in other languages into future research in order to obtain comprehensive results.

## CONCLUSION

This bibliographic analysis provides an overview of the machine learning application in ASD. Manuscripts investigating topics such as “Autism Spectrum Disorder”, “Machine Learning”, “pediatrics”, “electroencephalography”, “Diagnosis”, “deep learning”, and “Functional Connectivity” have attracted considerable attention. There are the following research directions: (1) A new field of investigation is human-in-the-loop and crowdsourcing machine learning for autism diagnostics; (2) Providing machine learning research with adequate support especially from employers or organizations; (3) Increasingly, doctors will be required to utilize tools and systems powered by artificial intelligence as health care moves to an era of intelligent technology. (4) Analyzing EEG signals for autism diagnosis especially in infancy is a good directions. During the coming years, machine learning technologies will contribute to the diagnosis of ASD and help clinicians.

In recent years, machine learning models have been applied to a wide range of problems. Because of the highly heterogeneous presentation of autism spectrum disorder, they may prove useful in early detection and could facilitate very early intervention.

## Figures and Tables

**Fig. (1):**
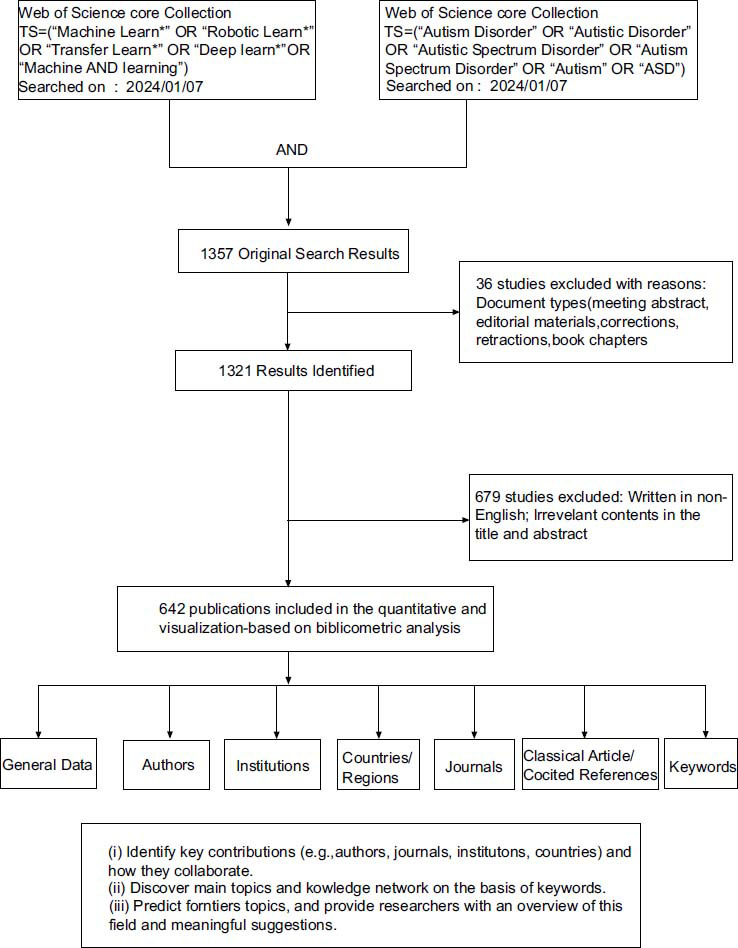
Analysis of bibliometric data and data screening flow chart.

**Fig. (3):**
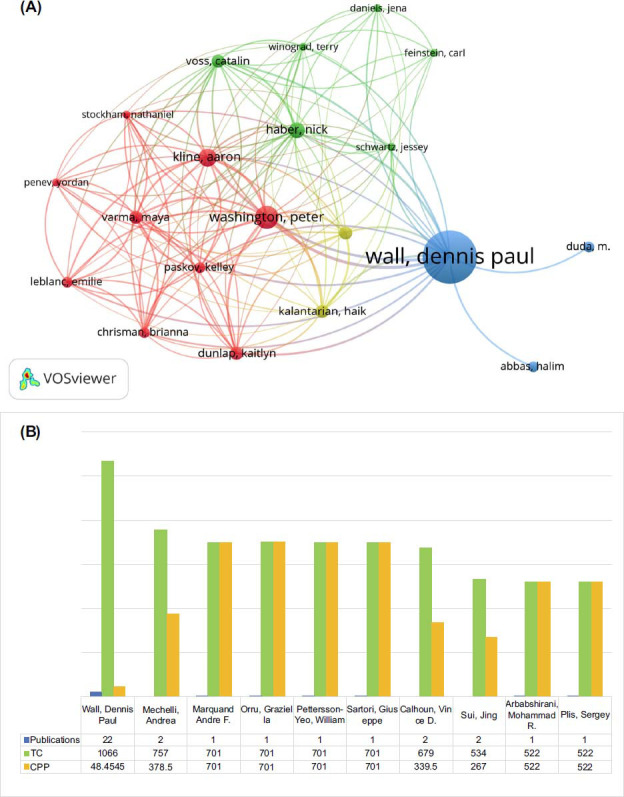
Collaboration network based on VOSviewer (**A**) between the most productive authors. The size of the node represents the number of documents. The width of the link represents the cooperation strength. (**B**) The top 10 most cited authors.

**Fig. (4):**
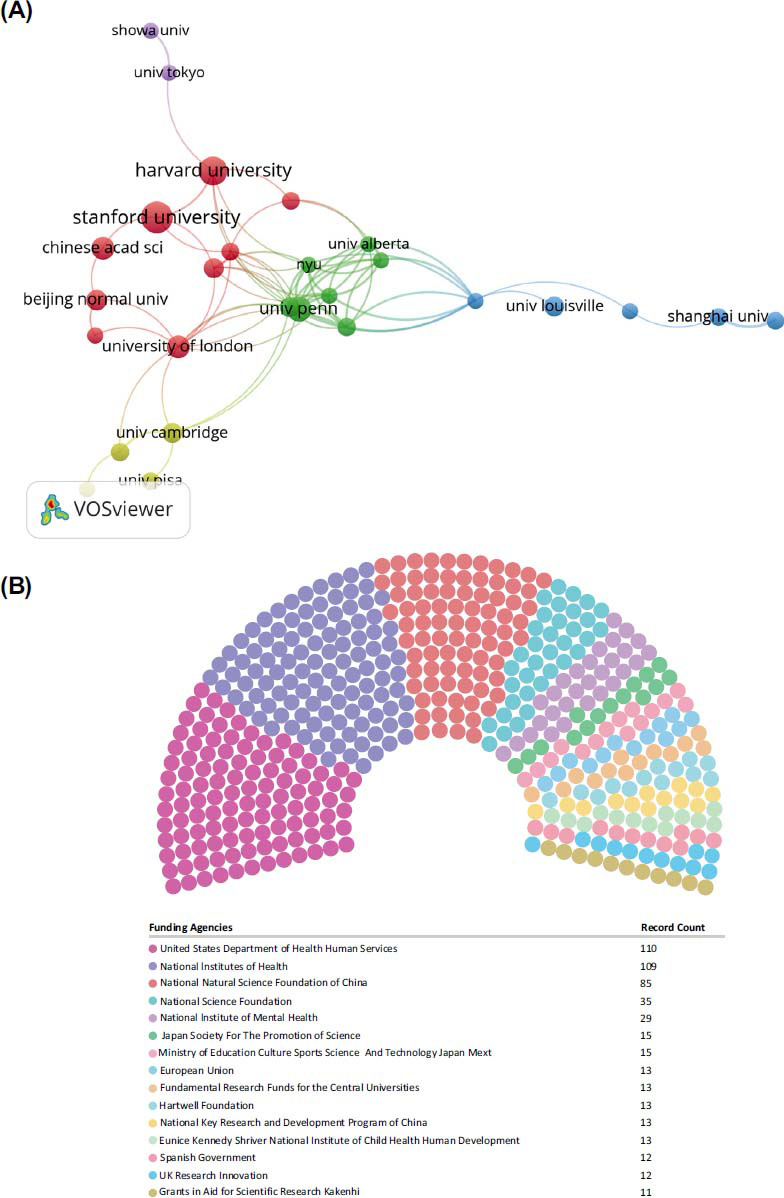
(**A**) Collaboration among institutions. A node's size indicates how many documents it contains. Cooperation strength is represented by the width of the link. (**B**) The top 15 most active funding agencies in machine learning in ASD.

**Fig. (6):**
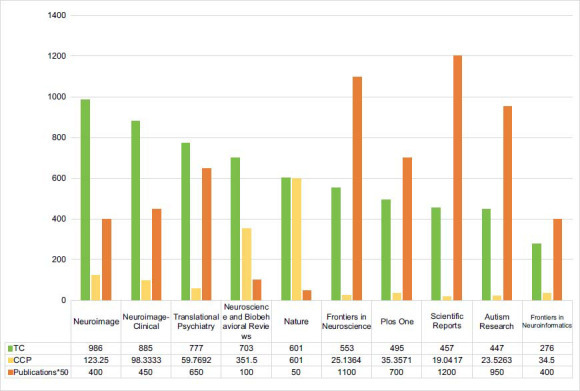
The top 10 most cited journals.

**Table 1:** Top 9 contributing Authors in machine learning in autistic disorder.

**Rank**	**Author**	**Publications**	**TC**	**CPP**	**Institution**
1	Wall, Dennis Paul	22	1066	48.4545	Stanford University
2	Thabtah, Fadi	12	394	32.8333	University of Huddersfield
3a	Elbaz, Ayman	9	91	10.1111	University of Louisville
3b	Washington, Peter	9	260	28.8889	Stanford University
4	Li, Jun	8	101	12.625	South China Normal University
5a	Calderoni, Sara	7	132	18.8571	University of Pisa
5b	Kline, Aaron	7	141	20.1429	Stanford University
5c	Xu, Lingyu	7	99	14.1429	Shanghai University
5d	Yu, Jie	7	99	14.1429	Chinese People's Liberation Army General Hospital

**Abbreviations**: TC, total citations; CPP, citations per publication.

**Table 2:** Top 11 prolific institutions of machine learning in autistic disorder.

**Rank**	**Institution**	**Publications**	**TC**	**CPP**	**Country**
1	Stanford Univ	26	955	36.7308	USA
2a	Harvard Univ	21	794	37.8095	USA
2b	Univ of California	21	534	25.4286	USA
3	Univ of Pennsylvania	16	1407	87.9375	USA
4a	Chinese Academy of Sciences	13	630	48.4615	China
4b	Univ of London	13	1018	78.3077	UK
5	Beijing Normal Univ	11	162	14.7273	China
6a	Children’s Hosp Philadelphia	10	992	99.2	USA
6b	Columbia Univ	10	126	12.6	USA
6c	Univ Cambridge	10	205	20.5	UK
6d	Univ Louisville	10	92	9.2	USA

**Abbreviations**: TC, total citations; CPP, citations per publication.

**Table 3:** The top 10 prolific journals related to ASD in machine learning.

**Rank**	**Journal**	**Publications**	**TC**	**CPP**	**IF(2022)**	**JCR (2022)**
1	Scientific Reports	24	457	19.0417	4.6	Q2
2	Frontiers In Neuroscience	22	553	25.1364	4.3	Q2
3a	Autism Research	19	447	23.5263	4.7	Q1
3b	Frontiers In Psychiatry	19	211	11.1053	4.7	Q2
3c	Ieee Access	19	196	10.3158	3.9	Q2
4	Plos One	14	495	35.3571	3.7	Q2
5	Translational Psychiatry	13	777	59.7692	6.8	Q1
6a	Biomedical Signal Processing and Control	12	54	4.5	5.1	Q2
6b	Brain Sciences	12	122	10.1667	3.3	Q3
7	Sensors	11	75	6.8182	3.9	Q2

**Abbreviations**: TC, total citations; CPP, citations per publication; IF, impact factor; JCR, Journal Citation Reports.

**Table 4:** Top 10 cited articles about machine learning in ASD.

**Rank**	**Title**	**First Author**	**Citation**	**Journal**	**Year**
1	Using Support Vector Machine to identify imaging biomarkers of neurological and psychiatric disease: A critical review [9].	Orrù Graziella	701	Neuroscience and Biobehavioral Reviews	2012
2	Early brain development in infants at high risk for autism spectrum disorder [20].	Hazlett, Heather Cody	601	Nature	2017
3	Single subject prediction of brain disorders in neuroimaging: promises and pitfalls [21].	Arbabshirani, Mohammad R	522	Neuroimage	2017
4	Identification of autism spectrum disorder using deep learning and the ABIDE dataset [22].	Heinsfeld, Anibal solon	416	Neuroimage-Clinical	2018
5	Genome-wide prediction and functional characterization of the genetic basis of autism spectrum disorder [23].	Krishnan, Arjun	230	Nature Neuroscience	2016
6	Functional neuroimaging of high-risk 6-month-old infants predicts a diagnosis of autism at 24 months of age [24].	Emerson, Robert W	201	Science Translational Medicine	2017
7	Functional connectivity classification of autism identifies highly predictive brain features but falls short of biomarker standards [25].	Plitt, Mark	190	Neuroimage-Clinical	2015
8	A small number of abnormal brain connections predicts adult autism spectrum disorder [26].	Yahata, Noriaki	174	Nature Communications	2016
9	Predictive models of autism spectrum disorder based on brain regional cortical thickness [27].	Jiao, Yun	170	Neuroimage	2010
10	Classification and prediction of brain disorders using functional connectivity: promising but challenging [28].	Du, Yuhui	157	Frontiers in Neuroscience	2018

## Data Availability

All data generated or analyzed during this study are included in this published article.

## LIST OF ABBREVIATIONS

ADHD: Attention Deficit Hyperactivity Disorder
ASD: Autism Spectrum Disorder
CAD: Computer-aided Diagnostics
CPP: Citations Per Publication
DTI: Diffusion Tensor Imaging
JCR: Journal Citation Report
SVM: Support-vector-machine
TC: Total Citations
